# Bridging barriers to advance multisector approaches to improve food security, nutrition and population health in Nepal: transdisciplinary perspectives

**DOI:** 10.1186/s12889-019-7204-4

**Published:** 2019-07-18

**Authors:** Santosh Gaihre, Janet Kyle, Sean Semple, Jo Smith, Debbi Marais, Madhu Subedi, Heather Morgan

**Affiliations:** 1 Division of Applied Health Sciences, University of Aberdeen, Aberdeen, UK; 2 Nutrition Innovation Centre for Food and Health (NICHE), School of Biomedical Sciences, Ulster University, Coleraine, BT52 1SA UK; 3 Institute for Social Marketing, Faculty of Health Sciences & Sport, University of Stirling, Stirling, Scotland; 4 School of Biological Sciences, University of Aberdeen, Aberdeen, UK; 5 Warwick Medical School, The University of Warwick, Coventry, UK; 6 Save the Children, Kathmandu, Nepal

**Keywords:** Transdisciplinary, Multisector, Intervention, Food security, Nutrition, Health, Low and middle-income countries

## Abstract

**Background:**

Understanding stakeholders’ perceptions is crucial to the development and implementation of any intervention. However, a structured approach to eliciting stakeholder insights into complex, multisector issues of food security, household environment and health is lacking in many low and middle-income countries. This qualitative, workshop-based participatory study explores stakeholders’ experiences of developing and implementing multisector interventions to provide transdisciplinary lessons for future developments in low and middle-income countries.

**Methods:**

Participants were purposely selected based on their involvement in, or exposure to, the multisector intervention. Participants with interests in agriculture, nutrition, household air-quality, drinking water-quality and health from academic institutes, government and developmental organisations were brought together at a one-day workshop to participate in a series of discussions on issues relating to food security, nutrition, household environment and health in Nepal. All group discussions were audio-recorded and transcribed, and a thematic qualitative analysis performed to identify relevant themes.

**Results:**

The government’s ongoing Multisector Nutrition Plan, stakeholders’ willingness to work together, availability of local infrastructure for cross-institutional inputs and increasing global movement towards transdisciplinary approaches were identified by the 33 workshop participants, representing 23 organisations as key factors determining success of transdisciplinary work. Fragmentation, lack of research-based and practice-based evidence, limited transdisciplinary knowledge amongst sectoral stakeholders, short-term funding and lack of knowledge-sharing mechanisms were identified as barriers, often creating systematic problems for successful implementation. Stakeholders suggested methods to bring about success included: improved knowledge, both amongst policy-makers and implementers, of food security and its linkage with nutrition, household environments, health and hygiene; investment in collaborative practice-based research and evidence-based practice; and strengthened transdisciplinary collaboration between multi-stakeholders, such as researchers, implementers and beneficiaries, throughout the intervention development and implementation process.

**Conclusions:**

This study suggests that multisector approach needs to adapt to take into account the experiences and views of the stakeholders concerned. The paper offers recommendations for successful development and implementation of future multisector interventions in Nepal that can be extrapolated to other low and middle-income countries, and lays foundations for future transdisciplinary working to support realisation of the recommendations.

**Electronic supplementary material:**

The online version of this article (10.1186/s12889-019-7204-4) contains supplementary material, which is available to authorized users.

## Background

Reducing food insecurity continues to be a priority in global public health. A wide range of agricultural and household interventions have been implemented in low and middle-income countries (LMIC) with varying degrees of success in terms of improving food productivity, nutritional status and health [1, 2]. Food insecurity, leading to under-nutrition, remains a major public health issue in many LMIC, with recent estimates suggesting that 805 million people in LMIC remain chronically undernourished [3]. In addition, those living in poverty in LMIC also suffer from high rates of illness from both communicable and non-communicable diseases that are linked to household air pollution [4, 5]; polluted drinking water; poor hygiene and sanitation; and a lack of appropriate medical care [6].

There is a growing awareness within academic, governmental and development organisations of the need to better understand the effectiveness of a transdisciplinary approach to tackle these complex and interlinked social, lifestyle and environmental problems [7]. However, there remain significant gaps in knowledge, including which interventions work well [8, 9] and whether packages of interventions, spanning multiple domains, work better than problem or condition-specific interventions [10]. The results of systematic review [11] highlight that complex packages of interventions to improve food security, household environment and health in a holistic manner show promise, but are currently rare and thus the effectiveness of such combined household interventions is not yet well understood. Furthermore, our review recommended a need to better understand the gaps, linkages and the factors influencing the development of complex interventions in LMIC. In addition, the contexts and mechanisms for implementation of complex interventions in real world settings need to be better understood.

Available evidence also suggests that knowledge of stakeholders’ perceptions and factors influencing user acceptability are crucial for design and effective implementation of any successful health promotion interventions [12–14]. A report by the United Nation (UN) high-level panel of experts (HLPE) on food security and nutrition also recommended exploring what can be done or changed within a multi-stakeholder partnership (MSP), by the partners themselves, to enhance the effective implementation of any interventions to improve food security, nutrition and sustainable development [15]. The HLPE report defines MSP as “any collaborative arrangement between stakeholders from two or more different spheres of society (public sector, private sector and/or civil society), pooling their resources together, sharing risks and responsibilities in order to solve a common problem or achieve a shared vision”. However, structured insights into transdisciplinary perceptions of public health and nutrition interventions are still lacking. Participatory and multidisciplinary workshops have been used to assimilate a wide range of information and to think through potential solutions for complex problems [16, 17]. Some have been shown to facilitate social learning, knowledge sharing, trust or relation building as well as enhancing participants’ understanding of cross-sectoral issues [12, 16]. However, despite the attention given to multi-stakeholder collaboration in knowledge generation, project planning and management, the commonly used mechanism of the participatory workshop has been given limited attention in the field of public health research, particularly in low-income settings.

For this study, Nepal, a low-income country, was selected as a case study to explore linkages and the factors influencing the development of complex interventions in LMIC using a multisector participatory workshop approach involving broad spheres of stakeholders. Nepal has varying levels of food insecurity (seasonal or annual) [18], and the majority of the people in rural Nepal have no access to safe drinking water and use biomass fuels for cooking and domestic heating [19]. As a result, many households suffer from food insecurity, malnutrition and ill health, not only due to poor diet but also from direct exposure to household air pollution, polluted drinking water, poor hygiene and sanitation [6].

The research reported in this paper was designed to address the following research questions:

1. What are stakeholders’ perceptions, knowledge and understanding of multisector approaches?
2. What are stakeholders’ perceptions of the factors influencing the development and implementation of successful complex interventions to improve food security, the household environment and health?

## Methods

### Study design and sampling

A multidisciplinary scientific workshop, led by the first author (SG), was held in Kathmandu, Nepal in November 2015, to qualitatively seek the views and opinions of a range of local stakeholders on the development and implementation of ‘holistic complex agricultural and household interventions’ in Nepal. Workshop participants included a range of stakeholders; scientific researchers, government officials, staff of national and international organisations working in Nepal and beneficiaries (farmers). A stakeholder-stratified, purposive sampling method was applied for participant recruitment [20]. Sampling started through discussion with key informants from each of the domains of interest for the study (food security, nutrition, the household environment and health) and/or target groups. Potential participants were identified and selected, explicitly targeting those stakeholders who were proficient in their field and likely to contribute to the generation of useful and appropriate information. Potential participants were then contacted by email and phone calls with the help of local key informants. A letter of invitation, explaining the aim and objectives of the workshop, and participant information sheets were sent to 63 potential participants including the health secretary and director general of the Ministry of Health in Nepal, experts at the National Nutrition and Food Security Secretariat of National Planning Commission of Nepal, Non-Governmental Organisations (NGOs), International Non-Government Organisations (INGOs) and local academics.

### Data collection

The workshop was opened with an introduction and presentations providing background to the aims of the session and results from a review of the literature [10]. The core activity of the workshop was facilitated group discussions. Participants were divided into five groups, each consisting of 6–8 participants, ensuring representation from each sector and stakeholder group within each group. A topic guide consisting of general themes constructed from the literature was developed and piloted to guide the discussion (see Additional file 1). Each group was given a single topic from the guide to discuss for 45 min. Group discussions were designed to be exploratory, and were structured around accompanying topics to address the research objectives. Five internal facilitators, who had previous experience working with diverse groups, acted as moderators for the group discussions. A pre-workshop meeting was held with the internal facilitators to inform them of the purpose of the workshop. All group discussions were audio-recorded and handwritten flip-chart notes including post-it notes were collected. Assurances of anonymity were addressed at the beginning of the group discussion to build trust with participants and an agreement to respect participants’ and organisations’ confidentiality. Group discussions were concluded with a self-selected representative from each group presenting the group’s notes to the larger group as a whole.

The workshop ended with an open, whole-group discussion and debriefing session, again allowing participants to put their views across on different topics, share their experiences and ask any questions. Additional insights and views generated during the open discussion were incorporated into flipchart notes.

### Data management

Each participant taking part in this study was given a unique study code for identification purposes, and was only linked to the participant through the Masterfile document. All quotes were anonymised to protect the identity of participants, but their role was denoted using sectoral labels and individual participants were numbered. All the records were kept in a secure storage area with access limited to research team members. All electronic data were stored in a password protected folder and stored on secure University of Aberdeen servers. Any personal data or information which could allow identification of individual study participants has not been presented in this paper.

### Analysis

Audio-records and flipchart notes were collated, transcribed verbatim into the local language, and translated into English, with quality checks from research team members (HM and JK). Each comment, quote and observation recorded from both audio transcripts and notes were subsequently categorised inductively in a matrix according to discussion topics in each group. Analysis [20] was based on the data captured in the matrix. After initial reading of the transcripts, the first author (SG) developed a manual colour coding system. This system identified initial patters and emergent themes across the data source. Summaries of the themes emerging from the data were entered into a Microsoft Excel matrix for further analysis. A thematic analysis method [21] was employed for the data analysis.

Themes and subthemes emerging within and across cases were identified by moving between the matrices and listening to the audio recordings and reading the transcripts. A further thematic analysis [21] was used to develop the typology categories and dimensions (SG supported by HM). The use of thematic analysis methodology helped to examine the local stakeholders’ perceptions on multisector intervention approaches, highlighting their experiences, knowledge, geo-political environment and availability of local resources based on the effects of a range of discourses operating within Nepal.

### Ethics

Ethical approval was obtained from the University of Aberdeen, College Ethics Review Board (CERB reference: CERB/2015/9/1239). All participants gave written consent for their participation, audio recording and the anonymised publication of quotes for research purposes.

## Results

**Fig. 1:**
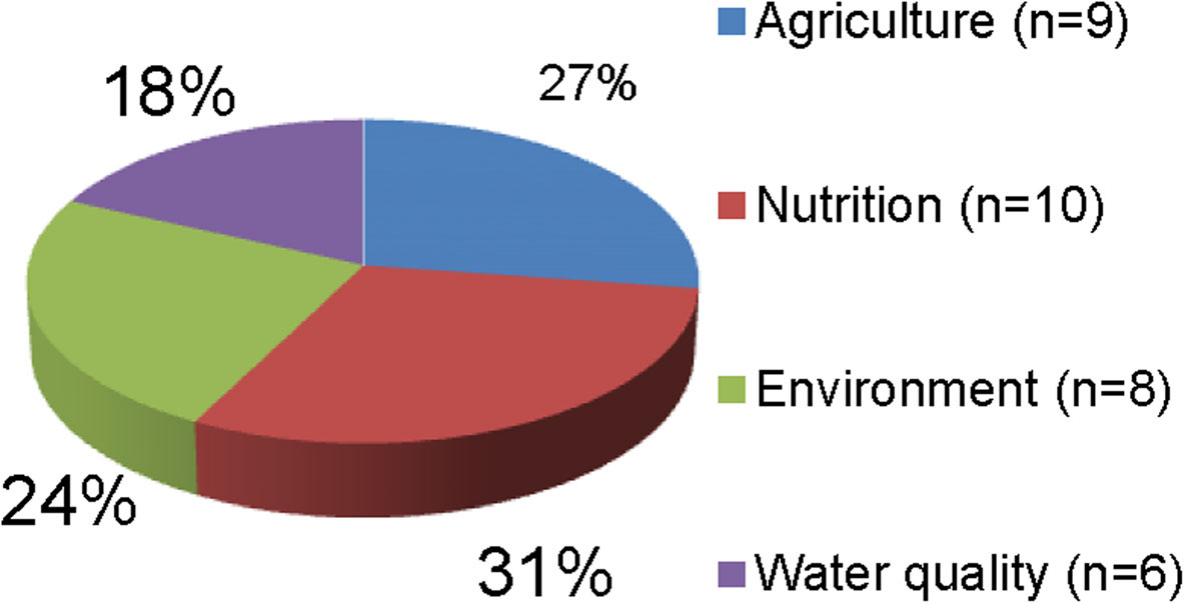
Participants by sector

**Fig. 2:**
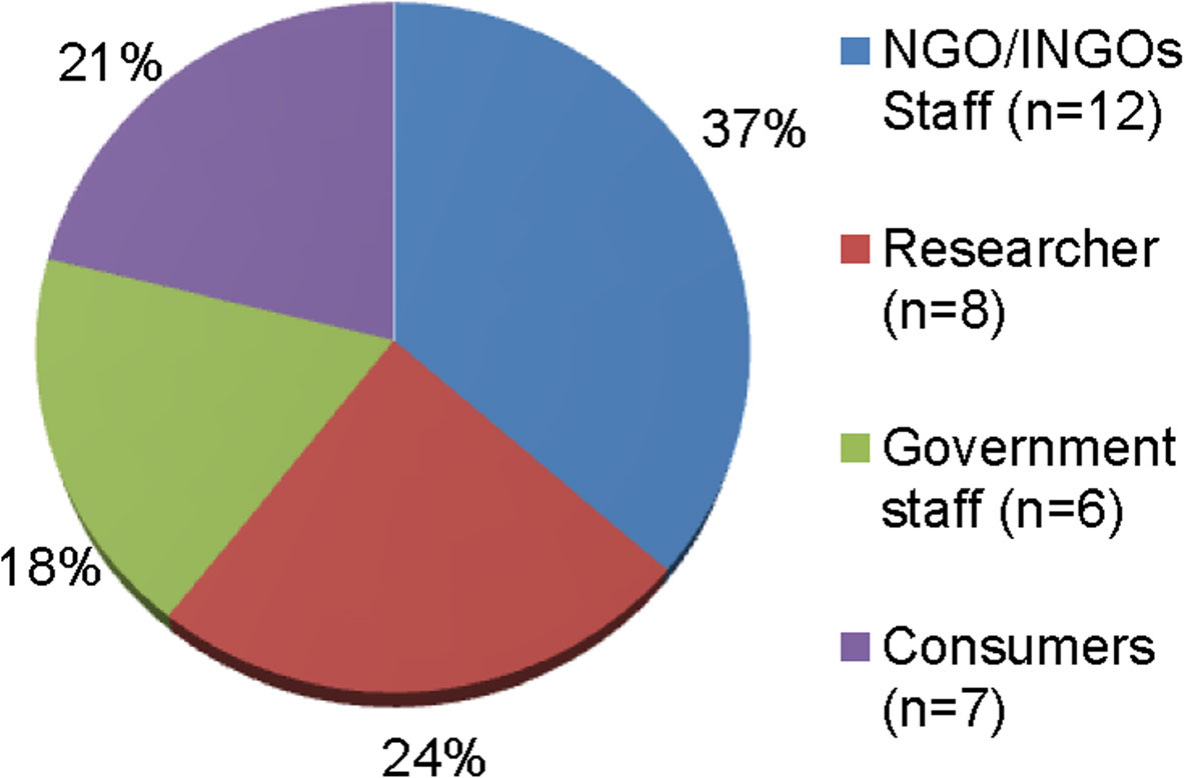
Participants by job role

Thirty-three stakeholders from 63 invitations attended the workshop (response rate 52%). Some people could not attend the workshop due to various reasons such as being out of town, other work commitments. Although the overall response rate could be considered low at 52% (although this was more than half), it is worth emphasizing that the use of a stakeholder-stratified purposive sampling method for participant recruitment ensured that the characteristics of those individuals who did participate in the workshop were similar to those who declined to participate. As such, workshop attendees represented agriculture (*n* = 9), nutrition and health (*n* = 10), environment (*n* = 8) and water quality (*n* = 6) sectors, and worked in government (*n* = 6), NGOs/ INGOs (*n* = 12) and academic institutions (*n* = 8) in Nepal (representing a total of 23 different organisations). In addition to that there was a good representation of the civil society (*n* = 7). The workshop was held at a hotel in central Kathmandu on Tuesday 10 November 2015. A breakdown of participants is shown in Figs. 1 and 2.

### Themes arising

Analysis of group discussions revealed the following main themes: local stakeholders’ knowledge and experience of multisector working; existing national policies and programmes; examples of good practices; research context; facilitators and barriers of programme implementation; and room for improvement.

### Knowledge and information

The majority of stakeholders were generally well informed about the benefits of multisector approaches and were aware of national initiatives including the Multisector Nutrition Plan (MSNP) [22], and *‘Sunaula Hazar Din’* Golden Thousand Days (GTD): Community Action for Nutrition [23]. Many participants commonly perceived multisector approaches as an innovative way to address complex public health issues in Nepal; “*Multisector nutrition plan is a milestone to start coordinated approaches in nutrition improvement in Nepal*….”(Government official 1).

The importance of existing knowledge and information to multisector household interventions aimed at improving overall heath was observed among the majority of the stakeholders, especially government and other development organisation representatives. From the discussion, it was observed that the current shift towards multisector initiatives and some capacity building programmes has already been initiated. It was mentioned that nutritional programmes for children in the past were domain-specific and targeted especially for the under-five age group; however, current policies are more integrated and focus on early life. For example, GTD initiatives, target the first 1000 days, starting from conception up to a child’s second birthday [23]. The level of knowledge and understanding of multisector approaches was relatively low among the project beneficiaries (i.e. community representatives) who requested more information of community awareness programmes. *“so many things are happening now and [we] don’t know which one is better…”* (Community representative 1).

### Tension between knowledge and practice

Despite recognising the benefits of multidisciplinary approaches, the majority of stakeholders, especially the government and developmental organisation representatives, reported that most of their work is still domain-specific. Although many of them clearly expressed their willingness to work together, they also highlighted the fact that there are no such established communication platforms to promote multisector approaches in Nepal. A feeling of lack of opportunities to choose the direction of work was also expressed. A government representative, for example, felt powerless to decide the direction of interventions in the context of donor-driven planning. He mentioned that most of their work is donor-driven [“*we are in fragmentation; most of our projects are donor driven, we haven’t done community need assessment. Although, I know I’m working for communities but most of our activities are responsible to donors not to the community…”* (Government official 2)], but highlighted some new initiatives of multisector approaches. Other participants discussed new programmes that include both nutrition and water, sanitation and hygiene (WASH) components, while other equally important factors, such as household air quality and climate change, are not incorporated in the MSNP. They also reported the need for government strategies to encourage multisector approaches and more funding and support for such initiatives.

### Existing national policy

Government policy and plans play a crucial role in the development and delivery of any local multisector programmes. A group of participants indicated that the management architecture (structure) of multisector approaches to improve food security, nutrition and health is already in place in Nepal. Various policy documents were discussed; among these, the *‘Multisectoral Nutrition Plan (MSNP)’* was the most commonly cited policy document throughout the workshop. The MSNP is a coordinated approach [22] of five key sectors; health, agriculture, education, urban development and local development to address the issue of nutrition in a systematic manner through implementation of nutrition specific and nutrition sensitive interventions. The MSNP is a unique plan within Nepal and across the world [22]. It has an established institutional mechanism to coordinate nutrition interventions from government policy making level (central level) to sub-national units at district, municipality and village level. It is being implemented through a series of cross-government governance structures, including those at ministerial level down to local level, facilitating engagement at all levels and an opportunity to engage with the various stakeholders. Many participants, mostly government and NGO/INGOs officials, were seen to have detailed knowledge of this policy and mentioned that it is in the initial phase of implementation. Some participants even highlighted the constraints and challenges of implementing the policy; they commented: “*it is time consuming, it has broad objectives, there is a lack of coordination among key government sectors and development agencies, limited budget allocation by each sectors and donors”,* etc. Gaps in the policy were also highlighted, emphasising what were considered to be the missing components. *“We have multisectoral nutrition plan in place, from top central level to the community (grassroots) level, which is good but still environmental components such as household air quality and climate change are not fully integrated in MSNP”* (Government official 3). However, outside government and NGOs, the extent to which the MSNP really led to cross-organisation action was widely questioned. It was notable that a common perception among the academics and researchers was that the plan was really a Ministry of Health initiative, with some work with other sectors. A researcher said they perceived MSNP as a Department of Health programme rather than cross-government initiative because it was always the health sector that coordinated nutrition-related plans and programmes.

While asking about the available evidence, participants mentioned that a portal for food security is in place and the Nepal government has already started documentation of available evidence to shed light on the status and trends in nutrition improvement. Similarly, a one door policy system for resource dissemination is already in place to facilitate the proper and equal distribution of resources. “*As different organisations implementing similar projects to improve food security, maternal and child nutrition and health, the government is aware of risk of resource duplication… and therefore, we (government) are trying to avoid the duplication at village and district levels. For example, if Golden Thousand Days are implementing in one district or village then the other similar projects such as SABAL [Sustainable Action for Resilience and Food Security] and SUAAHARA [Good Nutrition] are not going to be implemented in that area”* (Government official 2).

Participants also identified and summarised the key policies and interventions, and their achievements (Table 1).

**Table 1:** Summary of key policies and interventions to improve food security, nutrition, environment and health in Nepal

Food security interventions	Nutrition interventions	Environmental interventions
Guiding policies:		
• The Constitution of Nepal 2015 • Agriculture prospective plan (1995–2015) to enhance farmers’ capacity building • 13th Periodic National plan addressing food security • Food act and regulations • Agriculture development strategy • Nepal Food Security Monitoring System (NeKSAP)	• National health policy 2014, National nutrition policy 2004 • National health sector program (NHSP) 2nd until 2015 • Multisectoral Nutrition Plan 2012 • 13th periodic national plan • Sustainable Development Goals • Food and nutrition security plan	• Climate change policy (holistic policy linking health and environment) • National adaptation plan of action • National rural development strategy • National urban development strategy
Programmes:		
• Agriculture and food security project in 19 far western and mid-western districts • Agriculture programmes	• Promotion of maternal, infant and young child (MIYC) feeding from central to Village Developmental Committee (VDC) level • Vitamin-A and iron supplementation • Growth monitoring activities • School health and nutrition programmes • Behaviour Change Communication (BCC) programmes through media	• Multi stakeholder forestry programme

Overall, the majority of stakeholders in this subgroup, especially those working in government and developmental organisations, were aware of the current multisector policies to improve food security, nutrition, environment and health in Nepal. It was mentioned that existing policies and strategies, such as MSNP, climate change adaptation policy and national urban development strategies, are more holistic and link health and environment with other sectors. Some participants also mentioned that agriculture development strategy was developed in collaboration with the Ministries of Health and Agriculture to improve food security and nutrition status by running various community-based activities, such as livestock farming and seed improvement programmes. It was also discussed that the recent Constitution of Nepal 2015 has given recognition to food security by guaranteeing food sovereignty as a basic human right, but how to implement or achieve this was not clear to workshop participants. Although the effectiveness of these programmes at household levels has not yet been seen, for many stakeholders, improvement in existing policies gives the positive assurance that more emphasis has been placed on designing multisector collaborative programmes and projects. One participant noted that there was an *“overall increasing level of awareness amongst the project participants via various training, publications and capacity enhancement programmes”* (NGO representative 4). However, others perceived that while there are signs of improvement at the population level, changes at the household level remain to be seen, and these were considered to be the most important. “*There have been improvements in the chronic undernutrition, however, it is still high, as 41% of the children still suffer from chronic malnutrition and regional variations are also quite large… long way to go and we need more coordinated actions to achieve the national target”* (NGO representative 5).

Improvement in universal access to basic health services was highlighted as a key achievement by health sector representatives- “*Health facilities have been available at village level - we now have women health volunteers in each village ward level and are providing basic health services at peripheral level. We are also working with other sectors` to improve nutrition, sanitation and health- as a result there is a growing awareness amongst the village people about better nutrition, hygiene and sanitation. Some positive signs have already been seen- such as decreasing rate of communicable disease prevalence, polio eradication, decrease in maternal and child mortality rate and morbidity etc.”* (Government official 4).

### Key institutions responsible for change

The availability of workforce and resources, from both national and international sources, plays an influential role in shaping any interventions. Similarly, success of any policy and programme depends on the available infrastructure, coordination and cooperation between actors operating in different institutions and sectors. Having been asked to identify key institutions responsible to enhance multisector intervention in Nepal, participants identified and categorised the identified institutions into five main groups according to their type; government institutions, academic institutions, research institutions, training institutions, and national/international nongovernmental organisations (Annex 2). The main responsibilities and expertise of each institution were further explored during the data analysis and are summarised in a frequency table (Table 2) below. A detail typology of key institutions involved in the implementation of multisector intervention and programmes in Nepal are presented in additional file (see Additional file 2).

**Table 2:** Responsibility and expertise of the identified institution in multisector intervention in Nepal

Responsibility and expertise of the identified institutions	Frequency
Planning (P)	12
Implementing (I)	11
Monitoring (M)	15
Donor (D)	7
Capacity Building (CB)	6
Research (R)	7

Participants indicated that local infrastructure for cross-institutional inputs to run multisector programmes is available in Nepal. However, they also acknowledged the challenges of joint initiatives of government and development partners in multisector programmes. Lack of coordination among different organisations, their roles and linkages, duplication e.g. “*different institutions implementing similar programmes in one area”* (Researcher 1), overlaps, lack of continuation and sustainability were the common challenges highlighted by the local stakeholders. Lack of coordinated approaches on programme ownership; “*who will take the lead?”* (NGO representative 5), advocacy, awareness, knowledge dissemination; *“there is no clear mechanism for information sharing within and out of the organisation”* (Researcher 2) were also highlighted during the discussion. Some participants expressed additional concerns about the long term sustainability of multisector programmes and dominating characteristics of certain institutions, saying *“many of them [multisector programmes] are one off, no follow up or long term plan, most of them are donor driven and not in users or implementing bodies’ interest…”*(NGO representative 5). Good governance, active coordination, clear and transparent roles and responsibilities, sectoral reviewers, joint funding for review, and continuous sharing of knowledge, expertise and information within and between sectors were suggested as solutions to overcome these challenges.

### Examples of good practice

Participants highlighted some projects that are strong in terms of having multisector inputs. The GTD was one of those community-based nutrition projects frequently highlighted by this group and other participants in the workshop. The Nepal government in partnership with the European Union and United Nations Children’s Fund (UNICEF) [23] runs this project. The main aim of this project is to improve women’s access to essential nutrition services during pregnancy and until their children reach the age of two years. According to a government official, the Nepal government acknowledged that the first 1000 days of life, that is from conception to two years of age, is a unique window of opportunity for improving nutritional health. Therefore, a plan was implemented in 2012 to address the risk factors for chronic malnutrition, aligning with the main vision of the Nepal government’s MSNP. The government and NGO officials working in health and nutrition sectors were more in favour of this project and emphasised the importance of this project to shape child health in Nepal: **“***We know that 80% of child’s brain development occurs within the first 1000 days, therefore we called it ‘Sunaula Hazar Din’… Golden Thousand Day…”* (NGO representative 1). However, representatives from academic institutions and research organisations were unaware of this programme.

Another similar project mentioned by the participants was ‘*SUAAHARA’* (good nutrition) [24] to improve maternal, new-born and child nutrition health; this works to improve nutritional knowledge and household food security by increasing home grown foods, drinking water quality, hygiene and sanitation. Funded by USAID, the ‘*SUAAHARA’* project works closely with the government to strengthen policies and programmes that improve the health and nutritional status of women and children and is currently implemented in 20 out of 75 districts [24]. Although many participants praised *‘SUAAHARA’* as a multisector project, some participants think that climate change and environmental components are still missing from this project. *“SUHAARA is really a good project for food security, nutrition, health and sanitation but I think climate change and environmental components are being neglected on this programme…ammm.. May be I’m wrong, but I haven’t heard anything like this on this project”* (NGO representative 2).

*‘ANUKULAN’* (Community Resilience), a DFID UK-funded [25] integrated community resilience programme on agriculture, climate change, nutrition and income generation to address the impacts of climate change, such as drought and flood, was also highlighted in the workshop. Some participants suggested that *‘ANUKULAN’* is a good project; however, others raised the issue of missing household environment components, especially household air quality interventions. “*Majorities of poor and vulnerable people in rural areas are still depending on biomass as their main cooking fuel and therefore, it would be great to include clean fuel alternatives in this project*, *not only to improve HAQ [household air quality] but also to reduce deforestation”* (NGO representative 6).

### Existing gaps and problems in implementing multisector programmes in Nepal

**Fig. 3:**
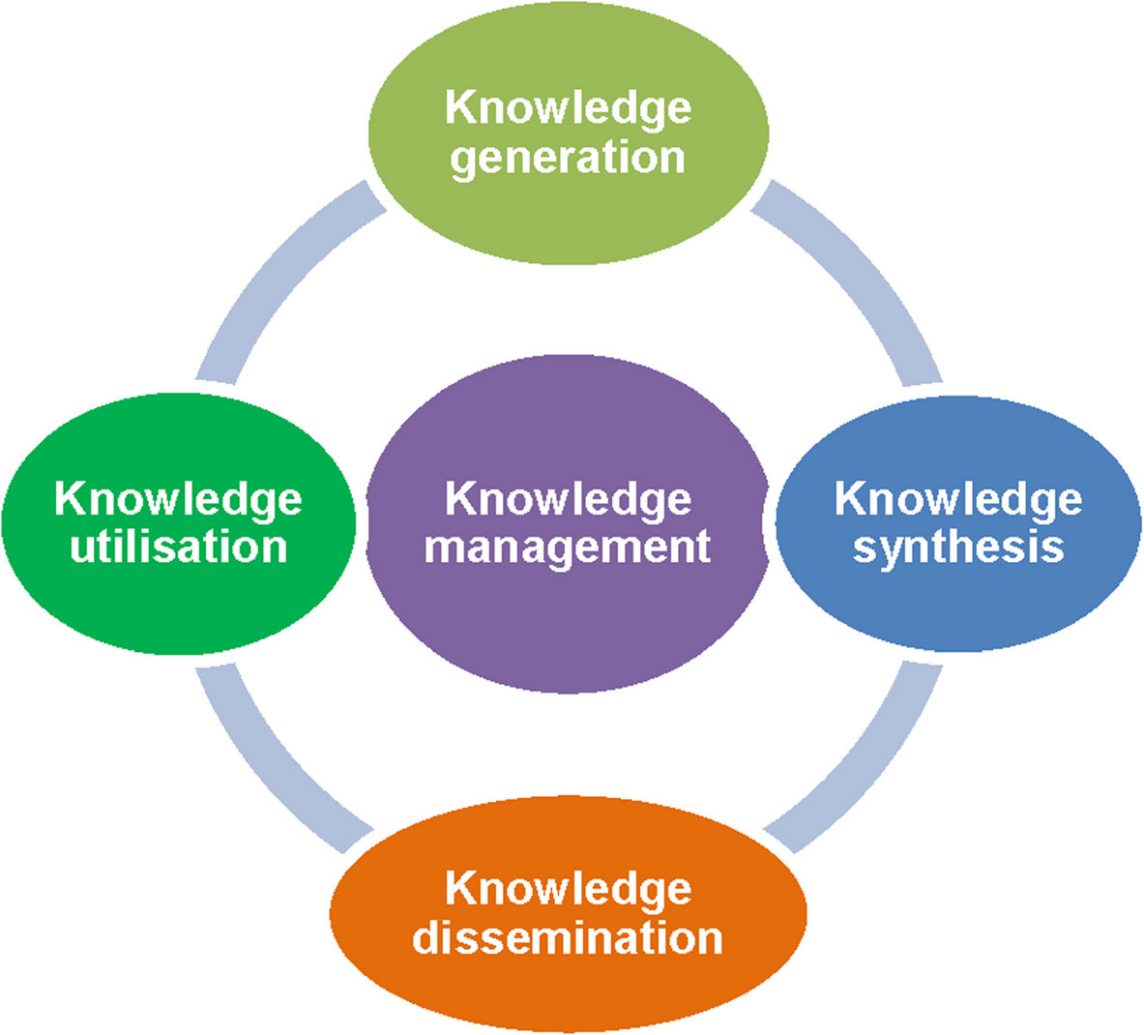
Knowledge management cycle

Participants discussed existing gaps in implementing multisector programmes in Nepal according to the following knowledge cycle (Fig. 3).

- **Knowledge generation:** Lack of scientific lab or field-based studies, such as plant breeding was discussed. “*Research related to iron content in wheats or rice hasn’t been done yet. Still we are giving iron and vitamin A supplementation to the pregnant women and children under five, don’t you think it would be good if they can grow iron or vitamin A rich wheats or rice themselves. Many countries have already started working in this line and our activities also need to focus on this issue”* (Researcher 3). Participants also highlighted the importance of understanding the food beliefs and behaviours of the local people before giving any advice regarding their use. The food behaviour and practices are strongly related to the cultural beliefs of a particular group of people living in different geographical areas of Nepal. For example stinging nettles (*sisno*), a widely available vitamin A rich leafy vegetable, is considered a poor people’s food in many part of the country. There is no proper evidence-based practice to break the barrier in food behaviours in different geographic areas to help the villagers make proper use of locally and cheaply available nutritious food; *“food behaviours and practices are different in different geographic areas and we cannot combine them in a single pot”* (NGO representatives 7).
- **Knowledge synthesis or packaging:** Nutrition charts need to be developed based on the local need and suitability for Nepalese households. For example, “*How much vitamin A is available in 2 mustard leaves? Or how many green leaves are required to fulfil the daily Vitamin-A requirement for a 6 months old complimentary feeding child. We have this information provided in the dietary reference intakes -microgram/dL- but that doesn’t make any sense for local farmers”.* (NGO representative 8)
- **Knowledge dissemination:** The right message and information is needed for the right target group, preferably in local language, via the right media, such as local radio, video or drama and social media.
- **Knowledge utilization:** Effective monitoring for corrective action and further learning is needed. “*We actually have resources but due to the lack of proper knowledge they haven’t been utilised properly…”* (Government official 6)

A need for practical local knowledge was also expressed.

*“While visiting our project sites villagers often ask some practical questions like this- It’s OK, I now understand green vegetables are good for my children. I have a one year child at home, so can you please tell me how many broad leaf mustard leaves I need to feed him daily to make him healthy?’…and I can’t give any answer….”* (NGO representative 8).

In addition, participants highlighted the lack of nutrition knowledge among the project beneficiaries.

*“In rural areas, some local farmers go to the nearby market to sell their home grown vegetables and use that money to buy processed-foods such as noodles for their children. They thought that fast foods are good for their children and children also like them most”.* (Community representative 2).

Myths and cultural issues, such as household food distribution patterns and women’s empowerment, also emerged during the discussion. One participant stated: “*Even pregnant and lactating women in rural areas still use traditional firewood stoves for cooking. There is a smoke problem, but she has no choice, and when time comes for eating, she has to feed the rest of the family members first and eats at the end whatever is left for her… this is ridiculous… and this sort of culture needs to be changed…”* (NGO representative 2).

Not reaching particular sections of the population was highlighted as another key problem in project implementation. “*We haven’t reached the vulnerable population yet, for example, Kapilbastu is one of the main food producing areas in Nepal but it also has severe nutrition problems, we haven’t reached those vulnerable populations with nutrition sensitive programmes, I’m hopeful MSNP will reach those areas in the near future”* (NGO representative 8).

Food insecurity, due to lack of appropriate policy and plans for food storage facilities, was also highlighted, giving the examples of persistence of high malnutrition rates in some areas despite sufficient food production: “*Well in some areas such as Kapilvastu district- there is sufficient food production but the trouble is that farmers can’t store their food for long term use… So not having proper food storage facilities is another challenge in many areas”* (Government official 5).

Similarly, the evidence-based knowledge gap was highlighted in the workshop, as well as a desire to integrate applied research in multisector plans and programmes. An NGO representative stated*: “Well… everything looks good on paper but we have no research-based knowledge particularly on food safety. We normally suggest people use a kitchen garden to grow varieties of green leafy vegetables for household consumption but we don’t have evidence-based knowledge whether it is safe to eat those vegetables especially when chemical fertilisers and pesticides were used in their garden”*. (NGO representative 4).

Lack of disaggregated information analysis for district-specific intervention design was highlighted by a NGO representative. *“We plan projects for Saptari (rural areas in southern Nepal) from Kathmandu (capital city) based on limited national level figures. For example, we use national level stunting prevalence rate of 41% to plan local level nutrition projects in all areas- we don’t have district specific data. There are large regional variations in the mid and far west hills and mountains in chronic malnutrition. So district and area specific data is needed to plan the right intervention for the right area”* (NGO representative 7).

A researcher mentioned inadequate technical capacity within available local service providers. “A *nutrition counsellor should be allocated at each health post”* (Researcher 5).

Workshop participants highlighted a lack of effective multisector coordination from central to the grassroots level. “*Integration needs to be at VDC [Village Development Committee] levels ‘When we want to run a focus group discussion related to nutrition and health issues in Dalits (the discriminated people) communities, VDC grant holders wouldn’t be willing to allocate their budget to support this programme, so where is integration?”* (NGO representative 6).

Participants also acknowledged the need for cost-effective programmes or cost-benefit analysis and effective monitoring of quality service and lack of capacity to make decisions under future uncertainty including natural disasters and climate change.

### Operation and implementation process

It was observed that the implementation infrastructure for the multisector plans do exist in Nepal. The National Planning Commission leads multisector coordination at various levels and develops evidence-based planning. The government representatives and some other NGO staff emphasised that the development and promotion of multisector ‘*bottom-up’* approaches is based on the 14-step annual planning process [26] of the government where the municipal and village development councils prepare three separate lists of projects based on the local needs. However, the community representatives did not agree with this. “*I live in a village which is not far from Kathmandu, and I’m an active community member of my village, but I’m just hearing about the role of community members on multisector project planning in this workshop… I haven’t seen any such activities in my area…”* (Community representative 3).

Resource mainstreaming was also highlighted as another major challenge for multisector programme operation and utilisation in Nepal. It was commonly agreed that there is disparity in resource allocation; *different sectors have different priorities and are allocating their budget based on their own sectoral priority.* In fact, participants mentioned that funding was a critical element in implementing multisector programmes, referring to both national and/or local funding. One of the NGO representatives said that funding was actually difficult to secure; that they had to fight with village development committees for what was available, suggesting a range of different experiences in practice.

The dominating nature of the sectoral (silo) working mentality among partner organisations was also highlighted as a big challenge for implementation and coordination of multisector programmes in Nepal. *“We still have sectoral working habits; most of the projects are still vertical e.g. breastfeeding, malaria, family planning, etc*. *Most of us were trained in a vertical working environment, which is creating a coordination problem. Health professionals are working from their own side and agriculture, nutrition and environment sectors working on their own priority areas. There is a multidisciplinary knowledge and resource gap… Health sector is not worried about environment, climate and agriculture”* (Researcher 4).

### Room for improvement in policy

Participants also expressed some suggestions and recommendations on current plans and policies and some ‘room for improvement’ themes emerged from their discussions. The researchers and representatives from academic institutions highlighted non-existence of interdisciplinary, transdisciplinary and collaborative research on multisector projects and programmes. Government officials and other participants were also concerned about this. Participant mentioned that there should be a balance between practice-based research and evidence-based practice: “*Policy should be based on research findings for example basic and local research...”* (Researcher 4). Some participants emphasised that research and dissemination of research findings need to be integrated and multidisciplinary. The need for inclusion of nutrition education in the school curriculum was also raised. Participants acknowledged the strong influence of teachers at school and identified the complementary roles of schools and education institutions in encouraging healthy diet and lifestyles. One of the participants said: “*Malnutrition and under nutrition programme should be integrated in school education curriculum so that children will be aware of it from their early years”* (Researcher 1).

There was a common voice within the group that while current plans and practices are good in the planning phase, implementation and monitoring phases were not satisfactory. Also, the common understanding was that the system needs money to improve itself but actually what this study suggests is that a genuine initiation to do things differently could bring a positive change. “*Planning and willingness is not enough you must initiate, initiation is not enough you must start, starting is not enough you must accomplish, accomplishment is always not enough you must ensure the quality, ensuring quality is always not enough you must make a difference, making difference is always not enough you must change, change is always not enough you must transform and transformation is need to be in beneficiaries [Golden Thousand Days mothers]. So, national vision on integrated perspective should be reflected in household levels. Such as WASH [Water, Sanitation and Hygiene], family planning, diversified food at home gardening, household environmental quality, improved cook stove to improve pregnant and lactating women and their babies’ health, etc. ‘Nobody is living in a perfect world- still there needs to be improvement’…”*(NGO representative 5).

Although very limited, multisector programmes are ongoing in Nepal; available examples suggest that the planning of ‘*bottom-up’* approaches (based on the 14 Step Annual Planning Process of Local Level, Nepal Government) is structured in Nepal, but there was consensus amongst local stakeholders that the monitoring and evaluation phase is still very weak. “*Monitoring framework is there but implementation of monitoring and evaluation framework is not there…. The indicators of monitoring and evaluation are there but system is not in place…”* (Researcher 4). As MSNP is currently rolling out, the national level planned evaluation has not been carried out. However, participants expressed their feelings about having a robust monitoring and evaluation procedure in place: “*monitoring should be done alongside with planning, but it is still too early to talk about national evaluation…”* (NGO representative 6)*.* There was agreement that the Nepalese government is good at planning, but weak on monitoring and evaluation, which needs to improve.

It was mentioned that a knowledge sharing platform is not available in Nepal. Many participants felt that having such a forum to share the best practice and lessons learned would be beneficial for them to increase multidisciplinary and transdisciplinary knowledge. It was also suggested that this could provide additional benefits such as networking opportunities for partners from various sectors to facilitate future coordination.

## Discussion

This study sought to qualitatively explore local stakeholders’ perceptions of the factors influencing the development and implementation of multisector household interventions to improve food security, nutrition, the household environment and health in Nepal. It explored the knowledge and understanding of multisector approaches amongst local stakeholders, as well as current practices, opportunities, facilitators, barriers and recommendations for ways forward for transdisciplinary working in Nepal with a view to providing lessons for other LMIC settings.

Knowledge and understanding of multisector approaches to improve food security, nutrition, household environment and health varied among the local stakeholders. Some discrepancy in knowledge could be due to sample composition rather than to opposing viewpoints of the participants. Although government and NGO representatives seemed aware of the multisector approach and some of them also had prior experience of working on multisector projects, the academic researchers and beneficiaries appeared relatively unaware of any ongoing local multisector and transdisciplinary initiatives. This revealed a general lack of functional collaboration between different actors as participants highlighted that not having any established knowledge sharing forum as an issue. Although there are many online knowledge sharing fora, such as SUN (Scaling Up Nutrition), Global Alliance for Improved Nutrition [27], WASH Plus (supportive environment for healthy community) [28] and Global Alliance for Clean Cookstoves [29], knowledge of and access to such online fora was very limited amongst the Nepalese stakeholders. This may be due to limited internet access and predominantly paper-based working cultures. Therefore, establishment of local knowledge sharing platforms to share lessons learned and best practices would be beneficial to increase transdisciplinary knowledge among local stakeholders. Similar recommendation was given by the UN’s high-level panel of experts on food security and nutrition emphasising that trust and synergies among partners can be preserved through continuous stakeholder engagement [15]. The participants also highlighted that the lack of multisector approaches is not due to limited budget and lack of knowledge about it amongst local stakeholders, but more due to the ‘silo mentality’ of donor-driven projects. Some government officials felt powerlessness with the donors and expressed a feeling of lack of the possibility of multisector work, despite understanding the benefits of it. A similar study by Kennedy and team also highlighted a lack of effective coordination between sectors and national to sub-national officials as a major concern for effective implementation of a multisector nutrition program to enhance nutritional status in Nepal [13].

In relation to existing policies, available evidence suggests that some policies and plans are already in place in Nepal to promote multisector approaches to address complex public health issues. For example, participants mentioned that MSNP [22], the Food and Nutrition Plan [30], the Ten-year Multi-stakeholder Forestry Programme [31], the National Rural Development Strategy [32] and the 13th Periodic National Plan are the holistic policies [33] linking agriculture, nutrition, health and environment. Nepalese stakeholders also reported some ongoing programmes such as GTD [23], *‘SUAAHARA* [24] and *‘ANUKULAN* [25]*,* as examples of multisector approaches. Although the overall perception of these policies, plans and programmes was very positive, some participants voiced criticisms of the implementation and evaluation mechanism. There was consensus amongst participants across all groups that they are good in planning (although a community representative did not agree with this), but weak in implementation, monitoring and evaluation practices. The findings of a systematic review on nutrition and the governance of agri-food systems in South Asia [14] also support this. They observed significant recent improvement in the development of policies and structures to strengthen the nutrition-sensitive agri-food system in the region, but highlighted a clear gap in terms of understanding implementation mechanisms and action on the ground [14]. Many participants also expressed their feelings about improvement on monitoring and evaluation, and expressed a desire for external support. There are many international organisations working in Nepal, such as DFID (The Department for International Development, UK), GIZ (the German Federal Enterprise for International Cooperation), USAID (US Agency for International Development) and the World Bank, which could bring expertise and experience on robust monitoring and evaluation. These organisations could help to fill this gap by providing technical support for monitoring and evaluation purposes; however, knowledge sharing and training for capacity building has not been forthcoming. This is not necessarily due to lack of goodwill or intention, but rather network infrastructure (e.g. lack of well-defined working protocols and practices).

Existing evidence suggests that effective multisector interventions can improve population health [34–36]; so it is likely that public health interventions will shift towards multisector approaches involving multi-stakeholder partnership, not just in Nepal or LMIC, but more broadly. However, lack of research-based evidence, poor planning, diverse services and working cultures, short-term funding, and limited technical and coordination expertise amongst local stakeholders do not provide systematic opportunities for multi-stakeholder partnership in multisector or transdisciplinary public health initiatives, with the exception of MSNP. A need for further research and effort to generate comprehensive evidence-based reliable information to enhance nutritional status is also emphasised on a recent UN report [15]. Existing evidence indicates that multisector approaches are popular and stimulate cross-sectoral engagement [37], lay the foundations for multisector action, and enhance knowledge and attitudes about the importance of transdisciplinary work [38]. This yields the insight that developing an intervention, which incorporates roles for all stakeholders and addresses the multiple determinants of health at a population level, can create political buy-in [39]. Basing the intervention on the best available local evidence and resources through practice-based research is necessary for its long-term sustainability. This indicates the need for a collaborative approach involving multi-stakeholder partnership with a focus on a specific area or objective.

A common understanding is that successful multisector approaches require established cross-institutional linking structures that promote shared understanding and accountability. It needs to be participatory and inclusive, with a clear role and responsibility for all partners involved and valued everyone’s contributions [12, 15, 40]. Multisector work also needs to be sustainable and time-bound, depending on purpose, for example sharing resources, problem solving, planning or implementing widespread change, and co-ordinating rather than duplicating efforts, clarifying responsibilities and lines of accountability in the system [41]. In addition to what was already known, this study added an exploration of local drivers, their responsibilities and current ways of collaborative working between different stakeholders, organisations and agencies, across sectoral and other boundaries in Nepal. This study confirms that local infrastructure for cross-institutional inputs to run multisector programmes is available and possible in Nepal; however, it may require changes to the usual ways of working and thinking.

Another insight is that establishing governance structure(s) for multisector interventions is necessary, but not sufficient to stimulate adequate cross-sectoral action. Cooperation is relatively easy where there are already shared agendas, such as shared priorities, funding and targets, but not where there are competing interests among different stakeholders or limited funding and resources [36]. Similar findings were observed in other research settings [42–44]. International experiences of successes and failures in multisector action in nutrition and public health suggest that time spent investing in evidence-gathering to identify shared goals, objectives and agendas may well produce better outcomes [45].

### Strengths and limitations of the study

The main strength of this study is the breadth of experience and expertise amongst the workshop participants, which provided unique contextual information and insights for the development and implementation of multisector approaches in Nepal, as well as for other LMIC. In addition, the layout of the group discussions made participants comfortable to share their views. However, some of the participants, especially government representatives working on the MSNP, may have been influenced by their personal involvement in it, so this should be considered in any interpretation of these findings. Although widely used in qualitative research, a purposive sampling method was used for participants’ recruitment, and may have influenced the findings.

## Conclusions

This study investigated the perceptions of stakeholders about the factors influencing the development and implementation of multisector approaches that could be used to inform the development of future transdisciplinary interventions to improve food security, nutrition, household environment and health in Nepal and other LMIC. It explored current links between agriculture, nutrition and environmental interventions in Nepal and also discussed facilitators and barriers identified by local stakeholders to advance the current practices. It was observed that policy and local infrastructure exists, and so does commitment to it, but improvement in ongoing monitoring and evaluation is needed. The Nepalese experience of the MSNP lends support to recommendations for improvement in policy, practice and research. The issue of insufficient interdisciplinary, transdisciplinary and collaborative research in this field was raised by stakeholders, who advised that research should work in balance with policy and practice so that there is evidence-based practice and practice-based research. Stakeholders suggested methods to bring about success included: improved evidence-based knowledge, amongst both policy-makers and implementers, to address underlying drivers of nutrition and health; investment in practice-based collaborative research; and strengthened transdisciplinary collaboration between multi-stakeholders, such as researchers, implementers and beneficiaries, throughout the intervention development and implementation process. Similarly, the need for knowledge sharing platforms and external technical support on monitoring and evaluation processes was expressed by local stakeholders. This study suggests that practice needs to adapt to take into account the experiences and views of the stakeholders concerned.

## Additional files

Additional file 1: Stakeholders' group discussion topic guide. (DOCX 12 kb)

Additional file 2: Typology of key institutions involved in the implementation of multisector programmes in Nepal. (DOCX 15 kb)

## Data Availability

The datasets supporting the conclusions of this article are included within the article and its additional files.

## Abbreviations

BCC: Behaviour change communication
CBOs: Community based organisations
CERB: College Ethics Review Board
DFID-UK: Department for International Development, UK
FAO: Food and Agricultural Organisation
GIZ: German Federal Enterprise for International Cooperation
GTD: Golden Thousand Days
HLPE: High level Panel of Experts
INGOs: International Non-Government Organisations
LMIC: Low and middle-income countries
MSNP: Multisector Nutrition Plan
NARC: National Agriculture Research Council
NAST: National Academy for Science and Technology
NeKSAP: Nepal Food Security Monitoring System
NGOs: Non-Governmental Organisations
NHRC: National Health Research Council
NHSP: National Health Sector Program
RECAST: Research Centre for Applied Science and Technology
SABAL: Sustainable Action for Resilience and Food Security
SNV: Netherlands Development Organisation
SUAAHARA: Good nutrition programme
UK: United Kingdom
UNEPA: United Nations Environment Protection Agency
UNICEF: United Nations Children’s Fund
USAID: US agency for International Development
VDC: Village Development Committee
WASH: Water, sanitation and hygiene
WFP: World Food Programme
