# Supplementary material for: Bridging barriers to advance multisector approaches to improve food security, nutrition and population health in Nepal: transdisciplinary perspectives
Source: BMC Public Health. 2019 Jul 18;19:961. doi: 10.1186/s12889-019-7204-4 (PMC6637542; doi:10.1186/s12889-019-7204-4)
Supplement: Supplementary file 1 — Stakeholders' group discussion topic guide. (DOCX 12 kb) [file 12889_2019_7204_MOESM1_ESM.docx]

1. Current practices of linkages among food security, nutrition, environment and health programmes in Nepal

2. Key Institutions involved in the implementation of multi-sectoral programmes related to food security, nutrition, environment and health in Nepal

3. Key interventions and achievements of multi-sector programmes on food security, nutrition, environment and health

4. Existing gaps in implementing multi-sector programmes involving food security, nutrition, environment and health in Nepal

5. Operation and utilisation procedures in implementing multisector programmes in Nepal
