# Supplementary material for: Bridging barriers to advance multisector approaches to improve food security, nutrition and population health in Nepal: transdisciplinary perspectives
Source: BMC Public Health. 2019 Jul 18;19:961. doi: 10.1186/s12889-019-7204-4 (PMC6637542; doi:10.1186/s12889-019-7204-4)
Supplement: Supplementary file 2 — Typology of key institutions involved in the implementation of multisector programmes in Nepal. (DOCX 15 kb) [file 12889_2019_7204_MOESM2_ESM.docx]

**Typology of key institutions involved in the implementation of multisector programmes in Nepal**

| Institutions | Responsibility |
| --- | --- |
| 1. Government institutions   Ministry of Agriculture Development  Ministry of Urban Development  Ministry of Health  Ministry of Population and Environment  Ministry of Education  Ministry of Federal Affairs and Local Development  Ministry of Science and Technology  Ministry of Finance  Ministry of Women, Children and Social Welfare  Ministry of Information Technology and Communication  Ministry of Home Affair  National Planning Commission  Human Right Commission  Courts  And their departments and related local offices | P, I, M, D  P, I, M, D  P, I, M, D  P, I, M, D  P, I, M  P, I, M  P, I, M, D, R  P, M, D  P, I, M  P, I, CB, M, ID  M  P, I, M  M  M |
| 1. Academic institutions   Different universities and their departments/faculties especially, environment science, microbiology, rural development, sociology, public health and various colleges | CB, R |
| 1. Research institutes   National Academy for Science and Technology (NAST)  National Agriculture Research Council (NARC)  National Health Research Council (NHRC)  Research Centre for Applied Science and Technology (RECAST) | R, CB  R  R  R |
| 1. Training institutions   National Soil Testing Centre  Centre for technical education and vocational training | CB, R  CB |
| 1. National and international nongovernmental organisations   United Nations bodies such as Unicef, World Food Programme (WFP), Food and Agricultural Organisation (FAO), UNEPA, NGOs, INGOs such as Save the Children, Oxfam, Plan International, Hellen Keller International, Practical Action UK, USAID, DFID (UKAID), Clean Energy Nepal, Centre for Rural Technology, SNP, ICIMOD and Community based organisations (CBOs).  Media; both local and national | D, P,I, M, CB    ID |

P= planning, I= implementing, M= monitoring, D= donor, CB= capacity building, R= research, ID= information dissemination
